# Altered cognitive function in systemic lupus erythematosus and associations with inflammation and functional and structural brain changes

**DOI:** 10.1136/annrheumdis-2018-214677

**Published:** 2019-04-12

**Authors:** Michelle Barraclough, Shane McKie, Ben Parker, Alan Jackson, Philip Pemberton, Rebecca Elliott, Ian N Bruce

**Affiliations:** 1 Arthritis Research UK Centre for Epidemiology, Division of Musculoskeletal and Dermatological Sciences, School of Biological Sciences, Faculty of Biology, Medicine and Health, The University of Manchester, Manchester, UK; 2 NIHR Manchester Biomedical Research Centre, Manchester University NHS Foundation Trust, Manchester Academic Health Science Centre, Manchester, UK; 3 FBMH Platform Sciences, Enabling Technologies & Infrastructure, FBMH Research & Innovation, Manchester Academic Health Science Centre, The University of Manchester, Manchester, UK; 4 Division of Musculoskeletal and Dermatological Sciences, School of Biological Sciences, Faculty of Biology, Medicine and Health, The University of Manchester, Manchester, UK; 5 Wolfson Molecular Imaging Centre, Institute of Imaging and Bioinformatics, The University of Manchester, Manchester, UK; 6 Department of Clinical Biochemistry, Manchester University NHS Foundation Trust, Manchester, UK; 7 Neuroscience and Psychiatry Unit, Division of Neuroscience and Experimental Psychology, The University of Manchester, Manchester, UK

**Keywords:** SLE, cognitive dysfunction, fmri, attention, stable disease

## Abstract

**Objectives:**

Cognitive dysfunction (CD) is common in systemic lupus erythematosus (SLE) but the cause remains unclear and treatment options are limited. We aimed to compare cognitive function in SLE and healthy controls (HCs) using both behavioural and neuroimaging techniques.

**Methods:**

Patients with SLE with stable disease and HCs were recruited. Clinical and psychological data were collected along with a blood sample for relevant biomarkers. Neurocognitive function was assessed using tests from the Cambridge Neuropsychological Test Automated Battery (CANTAB) and functional magnetic resonance imaging (fMRI) was used to examine brain responses to working memory (WM) and emotional processing (facial emotional recognition task, FERT) tasks.

**Results:**

Compared with HCs (n=30), patients with SLE (n=36) scored higher on measures of depression, fatigue and had higher hsCRP (p=0.013), IL-6 (p=0.003) and B lymphocyte stimulator (p<0.001). Patients with SLE had poorer performance on a task of sustained attention (p=0.002) and had altered brain responses, particularly in default mode network (DMN) regions and the caudate, during the WM task. Higher organ damage and higher VCAM-1 were associated with less attenuation of the DMN (p=0.005 and p=0.01, respectively) and lower BOLD signal in the caudate areas (p=0.005 and p=0.001, respectively). Increased IL-6 was also associated with lower BOLD signal in caudate areas (p=0.032).

**Conclusions:**

Sustained attention was impaired in patients with SLE. Poor attenuation of the DMN may contribute to cognitive impairments in SLE and our data suggest that in addition to mood and fatigue inflammatory mechanisms and organ damage impact cognitive functioning in SLE. The multifaceted nature of CD in SLE means any therapeutic interventions should be individually tailored.

**Key messages**

**What is already known about this subject?**

- Cognitive dysfunction (CD) is a significant problem in systemic lupus erythematosus (SLE), reported to affect up to 90% of patients.
- The cause is unclear and as such treatment is limited.
- No consistent measure of CD in SLE.
- Limited correlations found between structural brain abnormalities in SLE and cognitive function.
- Increasing interest in the use of functional magnetic resonance imaging (fMRI) in the assessment of cognitive function in SLE.

**What does this study add?**

- One of the largest fMRI studies in SLE.
- Assesses behavioural, functional and structural abnormalities in relation to cognitive function in SLE.
- Addresses the multifactorial nature of CD in SLE.
- Excludes patients with neuropsychiatric SLE (NPSLE) with the aim of examining the milder cognitive deficits reported by the majority of patients to address the concept of ‘brain fog’.
- Indicates that SLE-specific factors (e.g., inflammation, disease damage/duration) are associated with CD as well as chronic disease factors (e.g., fatigue, depression, pain, medication).

**Key messages**

**How might this impact on clinical practice or future developments?**

- Increases awareness that CD in SLE has multiple drivers and therefore treatment should be individually tailored.
- Helps in the development of a biomarker of CD in SLE.
- Aids future clinical trials by highlighting which key factors should be included in the study design.
- Highlights that patients with SLE are likely to employ compensatory brain mechanisms to maintain cognitive performance. As such patients may score similarly to healthy controls of objective measures of cognition but may fatigue quicker. This needs to be taken into consideration in any clinical trials or clinical assessments.

## Introduction

Cognitive dysfunction (CD) is one of the most commonly reported neuropsychiatric symptoms in patients with systemic lupus erythematosus (SLE) and significantly affects quality of life. While it has been reported in up to 90% of patients,^1^ treatment options remain limited in large part due to uncertainty around the cause(s), the lack of a consistent measure and the observation that patients with SLE may perform similarly to healthy controls (HCs) on objective testing.^2^

CD is common in other chronic conditions, such as inflammatory bowel disease and multiple sclerosis^3^ and factors associated with chronic disease such as mood disorders, medications and fatigue can all affect cognition. Specific SLE-factors are also hypothesised to play a role,^4^ with reported associations between autoantibodies and CD, particularly anti-*N*-methyl-D-asparate, anti-dsDNA and antiphospholipid (aPL) antibodies.^5^ Structural brain alterations in SLE such as cerebrovascular events, and the increased number of white matter hyperintensities^6^ also may contribute, although others have suggested that such structural changes are not directly associated with CD. As such, clinical and imaging biomarkers of CD in SLE remain elusive.

The American College of Rheumatology (ACR) established a recommended battery of cognitive tests but these require a trained professional to administer. An alternative is the Cambridge Neuropsychological Test Automated Battery (CANTAB), a tool that can assess changes in cognition over time, has been validated in many clinical settings, requires minimally trained administrators and has been successfully used in SLE.^7^ Another objective approach is functional magnetic resonance imaging (fMRI). fMRI gives a proxy measure of neuronal activation during cognitive testing. To date, only a few articles have reported fMRI findings in SLE but they suggest that patients with SLE may employ compensatory mechanisms within the brain to maintain adequate cognitive performance.^8^ Even fewer studies^9 10^ have examined cognition in SLE using a combination of behavioural, functional and structural assessments, although such an approach may better help identify causes and targets for therapy.

We aimed to compare cognitive function between patients with SLE with stable disease and HCs using CANTAB and fMRI. Variables that are known to affect cognition were also examined with differences between the two groups reported.

## Methods

Patients with SLE were recruited from Rheumatology departments at Manchester University NHS Foundation Trust Hospitals. HCs were recruited via study participants (e.g., friends) and social media. All SLE participants fulfilled ACR 1997^11^ or Systemic Lupus International Collaborating Clinics (SLICC) criteria^12^ for SLE and were considered clinically stable if no change of treatment was required, and their Systemic Lupus Erythematosus Disease Activity Index-2000 (SLEDAI-2K) score was ≤4.^13^ Participants with a history of epilepsy, stroke, severe depression/psychiatric conditions or certain central nervous system (CNS)-acting medications were excluded. Severe depression was defined as currently receiving treatment and/or scoring ≥20 on the Montgomery Asberg Depression Rating Scale (MADRS). Participants on low-dose CNS-acting medications or who were taking no more than three such medications (and only if being used to treat conditions other than depression, such as fibromyalgia) were included.

Disease activity was assessed using the British Isles Lupus Assessment Group Index BILAG 2004^14^ and SLEDAI-2K, and organ damage using the SLICC/ACR Damage Index (SDI).^15^ Specific biomarkers of the inflammatory response activation (B lymphocyte stimulator (BLyS), high sensitivity C reactive protein [hsCRP], interleukin 6 (IL-6)) and vascular/endothelial (vascular cell adhesion molecule-1 (VCAM-1), vascular endothelial growth factor (VEGF)) were measured.

All participants completed validated questionnaires on depression, anxiety and fatigue:

HADS: Hospital Anxiety and Depression Scale.^16^

BDI-II: Becks Depression Inventory-II.^17^

MADRS: Montgomery Asberg Depression Rating Scale.^18^

FSMC: Fatigue Scale for Motor and Cognitive Functions.^19^

After a literature review, we selected six CANTAB tests^20^ that assessed:

Visual memory and new learning (PAL: Paired Associates Learning).

Immediate and delayed verbal memory (VRM: Verbal Recognition Memory).

Emotional processing (ERT: Emotional Recognition Test).

Sustained attention (RVP: Rapid Visual Information Processing).

Executive function (OTS: One Touch Stockings).

Spatial working memory (SWM: Spatial Working Memory).

Two functional scans were performed using a 3.0 Telsa Philips *Gyroscan* ACS NT (Philips, Best, NL) MR scanner while participants completed a WM task (n-back) and a facial emotional recognition task (FERT). Two structural scans were also performed: a fluid attenuated inversion recovery and T1-weighted magnetisation-prepared rapid gradient-echo.

All behavioural and assessment data were analysed using independent t-tests for parametric data, Mann-Whitney U tests for non-parametric data and χ² for proportional data in SPSS 22 and group region of interest analyses were undertaken for the fMRI data using SPM12.

The target number of participants recruited to the study was determined based on fMRI power guidance, where a sample size of between 16 and 32 is considered acceptable.^21^

To examine any possible associations between SLE and CD exploratory Pearson/Spearman’s correlations and χ²s were undertaken using the SLE group only. These correlations were only conducted using the CANTAB tasks, structural brain abnormalities and fMRI results that were significantly different between the HC and SLE groups. These variables were assessed against factors proposed to affect cognition, including disease duration, disease activity, damage, medication use, aPL/LAC as well as measures of depression and fatigue.

Further details on all methods can be found in the online supplementary data.

10.1136/annrheumdis-2018-214677.supp1 Supplementary data

## Results

### Demographic and clinical findings

The SLE group were typical of a stable SLE cohort (table 1) and both groups were matched on age, gender, handedness and ethnicity; patients with SLE had fewer years in education and a lower mean IQ (table 2). The SLE group had higher depression scores (medians within the normal ranges). For each group, the percentages of participants that scored within the mild clinical ranges for depression/anxiety were: MADRS 0% HC, 6% SLE, BDI-II 6% HC, 33% SLE, HADS-D 3% HC, 39% SLE and HADS-A 20% HC, 39% SLE, despite excluding for clinical depression. The SLE group also had higher levels of motor and cognitive fatigue with median scores in the ‘severe’ (motor) and ‘moderate’ (cognitive) fatigue categories. Several biomarkers of inflammation and endothelial activation showed statistical difference between the two groups (table 2).

**Table 1:** Clinical and immunological characteristic of the SLE participants (n=36)

Characteristic	n (%) or median (LQ, UQ)
Female sex	34 (94%)
Disease duration (years)	10.5 (5, 15)
ANA positive (ever)	34 (94.4)
Elevated IgG anti-dsDNA antibody*	9 (26)
Low C3 or C4*	12 (35)
Anticardiolipin (aCL) antibody-positive*	9 (26)
Lupus anticoagulant positive*	6 (18)
BILAG total score†	1 (0, 2)
SLEDAI-2K	2 (0, 2)
SDI	0 (0, 1)
	9/36 (25%) had a score ≥1
Oral corticosteroids (y/n)	12 (33.3)
Average daily corticosteroid dose (mg) (n=12)	8.75 (6.25, 11.25)
Current immunosuppressant use	15 (41.7)
Current antimalarial use	22 (61.1)
Biological medication	3 (8.3)

*At time of study.

†Score calculated as stated in Yee *et al.* 22

ANA, antinuclear antibody;BILAG, British Isles Lupus Assessment Group Index;C3, complement component 3;C4, complement component 4;SDI, The Systemic Lupus International Collaborating Clinics/American College of Rheumatology Damage Index; IgG ds-DNA, immunoglobulin G double-stranded DNA.

**Table 2:** Demographic, psychiatric, fatigue and biomarker characteristics across the participant groups

Variable	SLE (n=36)	HC (n=30)	P value
	Mean (SD), Median (LQ, UQ) or n (%)		
*Demographic*			
Age (years)	40 (32, 48.75)	32 (27, 46.5)	P=0.14
Gender (% female)	34 (94)	30 (100)	P=0.19
Handedness (% right-handed)	30 (83)	28 (93)	P=0.34
Years in education	16.11 (3.51)	17.97 (3.40)	**P=** **0.034**
WTAR (IQ)	102.5 (98.25, 108)	111 (105, 114)	**p=** **0.001**
Ethnic origin			P=0.132
Caucasian	24 (66.7)	24 (80)	
Black Caribbean	4 (11.1)	0	
Black African	3 (8.3)	0	
Indian	1 (2.8)	0	
Bangladeshi	0	1 (3.3)	
Chinese	1 (2.8)	1 (3.3)	
Other	3 (8.3)	4 (13.3)	
*Depression*			
MADRS	4 (1, 8)	1 (0, 3)	**P=** **0.012**
HADS - D	4 (1, 9)	1 (0, 2)	**P<** **0.001**
BDI - II	10 (4, 20.25)	3 (0.75, 8)	**P=** **0.002**
*Anxiety*			
HADS – A	6 (3, 10.5)	5 (2, 7)	P=0.08
*Fatigue*			
FSMC – Motor score	36 (22, 40.5)	14 (11.5, 18.5)	**P<** **0.001**
FSMC – Cognitive score	31 (22, 40)	14 (11.5, 18.5)	**P<** **0.001**
FSMC – total score	67.5 (44.75, 80.5)	27 (23, 37)	**P<** **0.001**
*Biomarkers of inflammation and endothelial activation*			
hsCRP (mg/L)^1^	1.44 (0.66, 5.06)	0.88 (0.39, 1.39)	**P=** **0.013**
IL-6 (pg/mL)^1^	1.67 (0.50, 5.33)	0.50 (0.50, 1.32)	**P=** **0.003**
VCAM-1 (ng/mL)^2^	474.93 (194.30)	345.66 (53.79)	**P=** **0.001**
VEGF (pg/mL)^1^	66.04 (13.93, 139.60)	45.42 (6.04, 114.93)	P=0.275
BLyS (ng/mL)^1^	0.51 (0.35, 0.71)	0.34 (0.27, 0.39)	**P<** **0.001**

Missing data: WTAR not included for 3 HCs, and 4 SLEs, these participants’ first language was not English and/or they had dyslexia, as such it was felt that the scale would not accurately measure IQ in these participants. MADRS–5 SLE, 2 HC; FSMC–2 SLE, 1 HC hsCRP, IL-6, VEGF, BLyS-2 HC, 2 SLE; VCAM-1–2 SLE.

P-values in bold are significant at <0.05.

BDI-II, Becks Depression Inventory - II;BLyS, B lymphocyte stimulator; FSMC, Fatigue Scale for Motor and Cognitive Functions;HADS-A, Hospital Anxiety and Depression Scale–Anxiety score;HADS-D, Hospital Anxiety and Depression Scale–Depression score;IL-6, Interleukin 6;MADRS, Montgomery Asberg Depression Rating Scale;VCAM-1, vascular cell adhesion molecule-1;VEGF, vascular endothelial growth factor;WTAR, Weschler Test of Adult Reading; hsCRP, high sensitivity C reactive protein;.

All measures of depression positively correlated with both cognitive and motor scores of fatigue (FSMC) and negatively with years in education (see online supplementary table S1). In addition, hsCRP positively correlated with HADS-depression score (*r_s_*=0.43, p=0.013).

### CANTAB findings

From the 66 participants (36 SLE and 30 HC) who underwent CANTAB testing, 2 SLE participants did not complete all tests due to fatigue.

The SLE group performed less well on the RVP task (a test of sustained attention) compared with the HC group (13 [12, 20] vs 20 [15.75, 22], p=0.002). Compared with the normative data available from CANTAB 33.3% of the SLE participants scored one or more SDs below the RVP mean, whereas only 1% of HC group scored one or more SDs below the mean. The SLE group was also slower to identify emotions from the ERT task and identified more of the emotions incorrectly compared with HCs (p=0.012 and p=0.019, respectively) (table 3).

**Table 3:** Differences between the SLE and HC groups for each of the CANTAB outcome measures

Variable*	Measurement	SLE, n=36	HC, n=30	P value
		Mean (SD), Median (LQ, UQ), n (%)		
PAL+ (visual memory and new learning)	Total errors (adjusted)	29.50 (19.00, 79.75)	24 (10.75, 48.75)	P=0.095
VRM (verbal memory)	Free recall – total correct (Max.=18)	10 (8, 13)	10 (8.75, 14)	P=0.327
RVP (attention)	Total hits (Max.=27)	13 (12, 20)	20 (15.75, 22)	**P=** **0.002**
ERT (emotional processing)	Average percentage correct – total (%)	61.49 (8.85)	66.94 (9.36)	**P=** **0.019**
	Overall mean response latency – total (ms)+	1626.10 (1411.71, 2274.22)	1343.15 (1152.27, 1744.23)	**P=** **0.012**
OTS+ (executive function)	Mean choices to correct	1.40 (1.27, 1.73)	1.33 (1.18, 1.62)	P=0.484
SWM+ (working memory)	Between errors	108.41 (57.96)	94.73 (52.36)	P=0.328

Missing data: VRM: 1 SLE, ERT: 1 SLE; RVP: 1 SLE; SWM: 2 SLE; OTS: 1 SLE.

P-values in bold are significant at <0.05.

*Higher scores indicate better performance except where indicated with a “+”.

ERT, emotional recognition task; OTS, one touch stockings; Pal, paired associate learning; RVP, rapid information visual processing; SWM, spatial working memory; VRM, verbal recognition memory.

### Structural MRI findings

Structural analysis was conducted on 53 participants (30 HC and 23 SLE). The SLE group had significantly more and larger perivascular spaces (PVS) in the centrum semiovale (CSO-VRS), *χ^2^*=15.50, p<0.001. The differences between the SLE and HC group for the PVS in the basal ganglia (BG-VRS) did not reach significance, *χ^2^*=8.96, p=0.077 (see online supplementary tables S2-S4).

### Functional MRI findings

Not all patients underwent an MRI scan due to scheduling, discomfort and artefact issues. Overall, 23 SLE and 29 HC participants had fMRI scan data available for analysis.

#### n-back task results

Patients with SLE performed worse than HCs on the 0-back level (measure of attention, p=0.008) and were also slower to respond correctly on the 1-back and 2-back levels (measures of WM, p=0.019 and p=0.025, respectively) compared with the HC group (see online supplementary table S5).

##### Working memory condition (2-0back)

**Figure 1:**
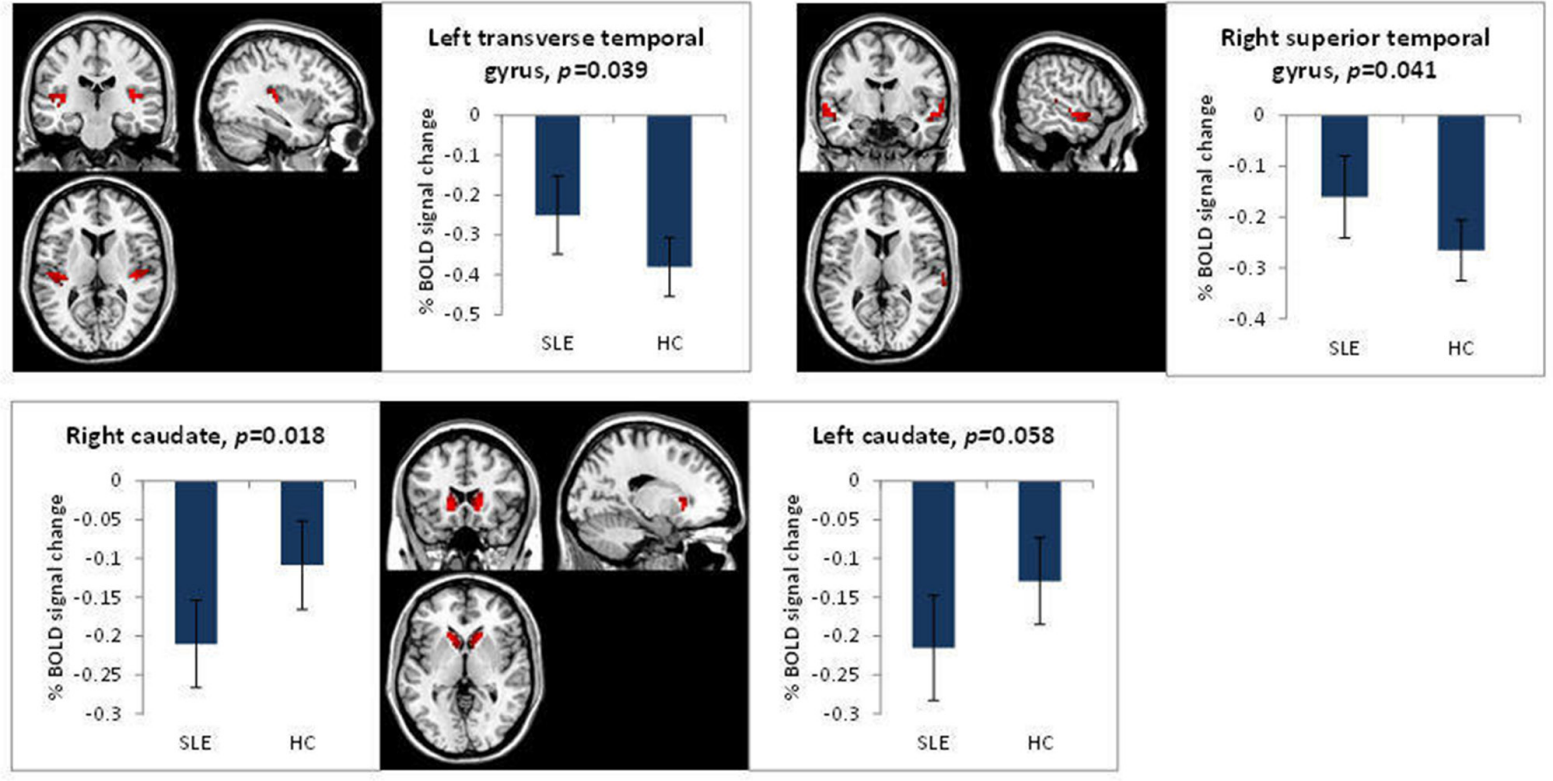
Significantly different BOLD responses for the SLE and HC groups for the n-back task, 2-0back, negative effect of task. HC, healthy control; SLE, systemic lupus erythematosus.

ROI analysis revealed significant results for the negative effect of the 2-0back condition. This condition highlights regions where the BOLD signal reduced for both groups during the WM task. Significant results were found in the left transverse temporal gyrus (*t*=2.12, p=0.039), right superior temporal gyrus (*t*=2.09, p=0.041) and right caudate (*t*=−2.45, p=0.018). The left caudate (p=0.058) also showed a similar BOLD response to the right caudate. Results in the left transverse temporal gyrus (LTTG-WM) and right superior temporal gyrus (RSTG-WM) showed a more decreased BOLD response for the HC group compared with the SLE group. In the caudate the reverse was found, the SLE group had a more significant decrease in BOLD signal (figure 1). No significant differences between the groups were found for the positive effect of the 2-0back condition.

##### Attention condition (0back-rest)

**Figure 2:**
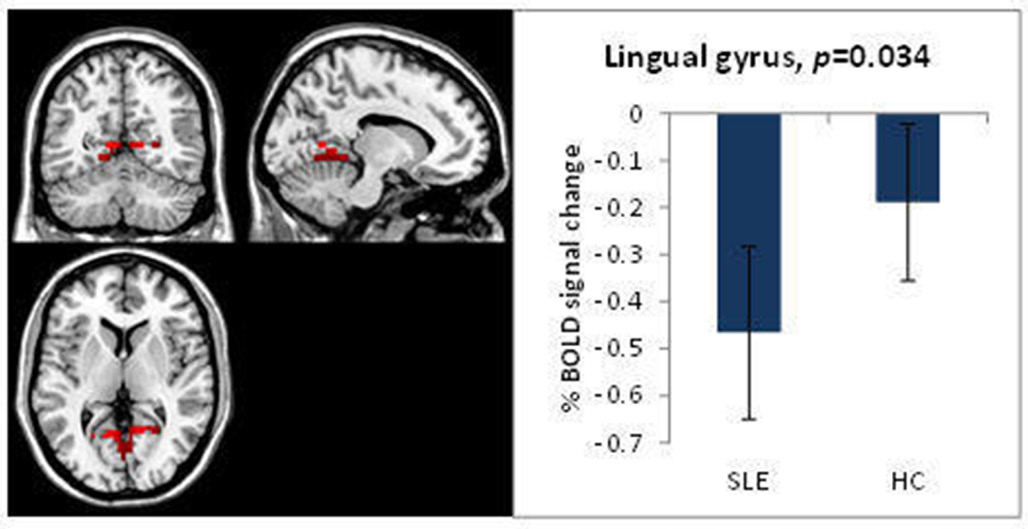
Significantly different BOLD response in the lingual gyrus for the SLE and HC groups, for the n-back task, 0back-rest, negative effect of task. HC, healthy control; SLE, systemic lupus erythematosus.

Using ROI analysis, the SLE group had a greater decrease in signal in the lingual gyrus compared with the HC group (figure 2). No significant differences between the groups were found for the positive effect of the 0back-rest condition.

#### FERT task

**Figure 3:**
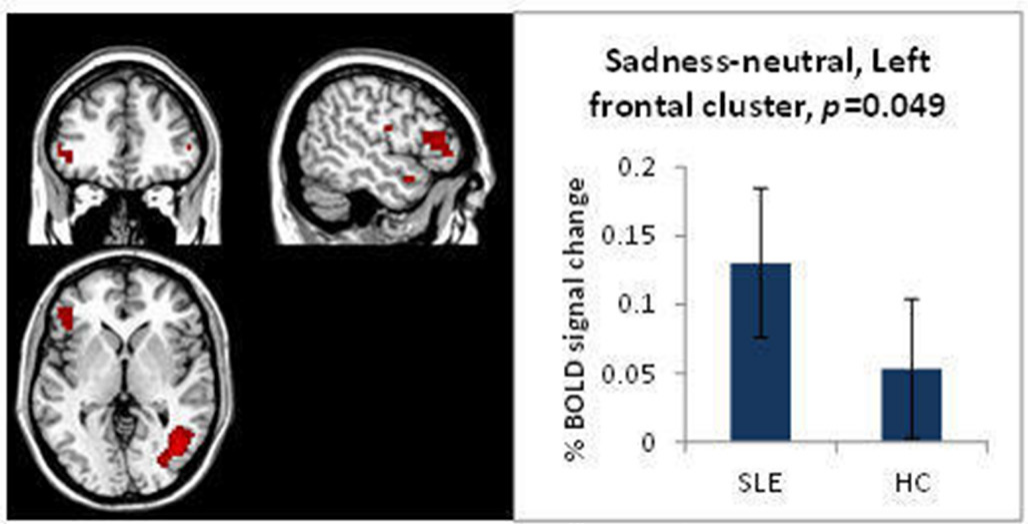
Significantly different BOLD response for the FERT, sadness-neutral, positive effect of task, SLE vs HC. FERT, facial emotional recognition task; HC, healthy control; SLE, systemic lupus erythematosus.

Behaviourally, the SLE group was slower to correctly determine whether a face was female or male when displaying sadness (p=0.035) (see online supplementary table S6). In an ROI analysis, in the sadness-neutral condition, the positive effect of task for this condition showed that the SLE group had an increased BOLD response in frontal areas compared with the HC group (figure 3). There were no differences in the BOLD responses for the negative effect of task.

### Exploratory associations between SLE and cognitive function

Improved performance on the attention task negatively correlated with the signal in a default mode network (DMN) region during the WM task (RVP associated with the RSTG-WM, *r*=−0.60, p=0.003).

Better performance on the emotional processing task negatively correlated with the signal in a DMN area during a WM task (ERT average percentage correct associated with the RSTG-WM, *r*=−0.72, p<0.001). Also, the mean response latency for the identification of the emotions in the emotional processing task negatively correlated with the signal in a cognitive region during the WM task (ERT overall mean response latency associated with left caudate-WM, *r*=−0.51, p=0.013), implying a greater response in the DMN when performing better and quicker on an emotional processing task.

Structurally, the enlarged PVS in the centrum semiovale (CSO-VRS) and basal ganglia (BG-VRS) were associated with years in education (*r_s_*=−0.48, p=0.022 and *r_s_*=−0.45, p=0.035, respectively). Neither was associated with vascular biomarkers including LAC or aCL positivity, or VCAM-1.

The DMN areas (left transverse temporal gyrus and right superior temporal gyrus), areas that are usually attenuated during cognitive tasks, were positively correlated with depression (LTTG-WM with MADRS, *r*=0.45, p=0.036), VCAM-1 (with RSTG-WM, *r*=0.53, p=0.01), SDI (with RSTG-WM, *r_s_*=0.56, p=0.005) and current use of biological medication (with RSTG-WM, *r*=0.60, p=0.003). The cognitive areas (right and left caudate) were negatively correlated with IL-6 (with the right, *r_s_*=−0.47, p=0.032), VCAM-1 (with the left, *r*=−0.65, p=0.001), SDI (with the left, *r_s_*=−0.57, p=0.005), disease duration (with the left, *r*=−0.49, p=0.019) and current use of biological medication (*r*=−0.52, p=0.011). The left caudate was positively correlated with cognitive fatigue as measured by the FSMC-Cognition score (*r*=0.43, p=0.047). Also, the right caudate was positively correlated with aCL positivity (*r*=0.51, p=0.015) but not with LAC positivity.

The attention condition of the n-back task, in the visual attention region of the lingual gyrus, positively correlated with the BILAG total score (*r_s_*=0.45, p=0.033), IL-6 (*r_s_*=0.44, p=0.036), current use of immunosuppressant (*r*=0.48, p=0.019) and antimalarial medication (*r*=0.47, p=0.028) and negatively with BG-VRS score (*r_s_*=−0.46, p=0.030) suggesting that higher inflammatory disease activity increased responses in an attentional brain region during an attention task.

The BOLD response to sad faces from the FERT task in the left frontal cluster negatively correlated with the SDI score (*r_s_*=−0.57, p=0.005) and disease duration (*r*=−0.43, 0.047).

## Discussion

We have identified structural, cognitive and fMRI differences in patients with SLE. While overall cognitive function was comparable between groups, the SLE group was less accurate on a test of sustained attention. Anatomically, we found increases in the PVS in the centrum semiovale in 43% of SLE participants and no controls. Using task-based fMRI, there was significant interference in emotional tasks and a reduced ability of patients with SLE to suppress the DMN during cognitive tasks.

Our data support previous work showing that attention is the most common cognitive problem in SLE.^4^ Such attention deficits can result in problems with other cognitive functions, such as WM although in this study we did not find any other non-emotional cognitive problem in the SLE group. A more detailed assessment of these relationships was limited due to time constraints with testing; however, follow-up studies focusing on these key inter-relationships are planned.

The 0back-rest condition of the fMRI n-back task is a neuronal marker of sustained attention. Patients with SLE had a larger task-negative BOLD response in the lingual gyrus compared with the HC group. This region has been associated with visual attention, visual encoding/processing and WM.^23–25^ This may explain why our SLE group performed worse on the behavioural (CANTAB) attention task. The few studies published using fMRI in SLE have suggested that patients with SLE employ compensatory brain mechanisms to maintain cognitive function.^8^ Our cohort may have failed to recruit compensatory mechanisms on the challenging sustained attention task, resulting in performance deficits. Exploratory analyses also found that the response to attention in the lingual gyrus negatively correlated with an increase in PVS in the basal ganglia. Previous studies have found patients with SLE to have a greater number and larger PVS in the basal ganglia which did not reach significance in our study; we did however find differences between the HC and SLE in PVS in the centrum semiovale. PVS is an imaging marker for cerebral small vessel disease;^26 27^ however, we did not find any correlations between several serological markers of vascular disease (LAC, aCL antibodies and VCAM-1) and CSO-VRS, although we did exclude patients with severe vascular disease.

On the CANTAB battery, there were no differences between groups for the behavioural WM tasks. However, on fMRI patients with SLE had less task negative BOLD signals in the left transverse temporal gyrus and right superior temporal gyrus. These areas are part of the DMN, which is usually inactive during cognitive tasks^28^ and active during rest and internal processes, such as self-reflective processes and planning.^23 28^ The limited ability to reduce these signals in SLE implies an inability to inhibit self-reflective processes which can impede performance on cognitive tasks that do not usually have an emotional component, by allowing emotional interference from self-reflection and worries about task performance.^29^ In support of this, the FERT fMRI task found that patients with SLE had a greater response to viewing sad faces in frontal regions compared with the HC group. Such increased responses to sad expressions is also associated with depression.^30–32^ Similarly, patients with SLE were less accurate in correctly identifying emotions on CANTAB and showed evidence of reduced response latency implying a level of psychomotor slowing, both of which are associated with depression^32 33^ and may contribute to some of the differences observed between groups. This is despite our groups scoring within normal ranges on the depression scales and that major depression was an exclusion criteria. We therefore cannot rule out the potential impact mood may have on cognitive performance in this SLE group even at the subtle end of the scale. Also, we recruited a low disease activity cohort and excluded NPSLE cases, so there may be different subtypes depending on CD severity.

We also noted differences in the caudate between the two groups. Patients with SLE had a larger task negative response compared with the HC group. The caudate, via the network linked to the dorsolateral prefrontal cortex, has been implicated in WM^34^ but usually as an area with an increased BOLD response during WM tasks. The attenuated response we found is in contrast to Mak *et al* who noted, using a different executive function task, an increased BOLD response in the caudate body from patients with SLE.^35^ It is therefore unclear if the differences we found are task specific and why our findings were in a task negative direction.

As noted, the SLE group had significantly higher scores on scales for depression and fatigue. While depression scores were still within the ‘normal range’ both motor and cognitive fatigue scores were higher in the SLE group. Both fatigue and mood can affect cognition^36 37^ and these symptoms are highly prevalent in SLE populations. In our study, mood and fatigue negatively impact neurocognition. It is increasingly recognised that inflammation and mood are closely interlinked.^38^and we found that VCAM-1, IL-6 and BILAG 2004^14^ scores correlated with cognitive brain mechanisms, supporting the hypothesis that inflammation in SLE contributes to CD. We also noted associations with the SDI and disease duration, strengthening the suggestion that specific SLE factors directly impact on cognitive function over time.^39^

We acknowledge some limitations to this study. For the non-fMRI analysis, the participant numbers are small; due to strict exclusion criteria and the use of fMRI as the main outcome. We also made no adjustments for multiple comparisons. However, many factors were closely correlated and in an exploratory study such as this, a Bonferroni correction would be too conservative. The HC group had a higher IQ and slightly more years in education than the SLE group. IQ can affect performance on cognitive tests but for many of the cognitive measures, no differences were seen so it is unlikely that this was the case. It was also impossible to recruit a SLE patient group on no medication. Patients on low dose psychoactive medications were included as well as those on corticosteroids. The correlations within the SLE group found no significant associations between corticosteroid dose and cognitive measures. Lastly, we chose to use the CANTAB battery as it is a sensitive measure of cognitive function that can assess changes over time and is easy to administer; however, some research has suggested that the tests measure overall cognition but cannot be divided into specific domains, such as executive function, so caution may be needed when interpreting individual test results.^40^

In patients with SLE, we have noted impairments in sustained attention while other non-emotional cognitive functions remained unaffected. Poor attenuation of the DMN may contribute to CD in SLE, although prospective studies may be needed to confirm this, and our data suggest that in addition to mood and fatigue, inflammatory mechanisms and organ damage impact cognitive functioning in SLE.

Clinically, this study has implications when advising patients about CD in SLE. It has highlighted the multifaceted nature of CD in SLE and that future therapeutic approaches will need to be individually tailored to address the relevant drivers in individual patients.

## References

[1] Tomietto P, Annese V, D'agostini S, et al General and specific factors associated with severity of cognitive impairment in systemic lupus erythematosus. Arthritis & Rheumatism 2007;57:1461–72. 10.1002/art.23098 18050188

[2] Hanly JG, Omisade A, Su L, et al Assessment of cognitive function in systemic lupus erythematosus, rheumatoid arthritis, and multiple sclerosis by computerized neuropsychological tests. Arthritis Rheum 2010;62:1478–86. 10.1002/art.27404 20155829 PMC4656033

[3] Attree EA, Dancey CP, Keeling D, et al Cognitive function in people with chronic illness: inflammatory bowel disease and irritable bowel syndrome. Appl Neuropsychol 2003;10:96–104. 10.1207/S15324826AN1002_05 12788684

[4] Kozora E, Hanly JG, Lapteva L, et al Cognitive dysfunction in systemic lupus erythematosus: past, present, and future. Arthritis Rheum 2008;58:3286–98. 10.1002/art.23991 18975345

[5] RC H, Thiaghu C, Ong H, et al A meta-analysis of serum and cerebrospinal fluid autoantibodies in neuropsychiatric systemic lupus erythematosus. Autoimmun Rev 2016;15:124–38. 26497108 10.1016/j.autrev.2015.10.003

[6] Luyendijk J, Steens SCA, Ouwendijk WJN, et al Neuropsychiatric systemic lupus erythematosus: lessons learned from magnetic resonance imaging. Arthritis Rheum 2011;63:722–32. 10.1002/art.30157 21360502

[7] Calderón J, Flores P, Babul M, et al Systemic lupus erythematosus impairs memory cognitive tests not affected by depression. Lupus 2014;23:1042–53. 10.1177/0961203314536247 24879658

[8] Barraclough M, Elliott R, McKie S, et al Cognitive dysfunction and functional magnetic resonance imaging in systemic lupus erythematosus. Lupus 2015;24:1239–47. 10.1177/0961203315593819 26124237

[9] Kozora E, Ulug AM, Erkan D, et al Functional MRI of working memory and executive dysfunction in systemic lupus erythematosus and antiphospholipid antibody positive patients. Arthritis Care Res 2016. 10.1002/acr.22873 26946337

[10] Shapira-Lichter I, Vakil E, Litinsky I, et al Learning and memory-related brain activity dynamics are altered in systemic lupus erythematosus: a functional magnetic resonance imaging study. Lupus 2013;22:562–73. 10.1177/0961203313480399 23535531

[11] Hochberg MC Updating the American College of rheumatology revised criteria for the classification of systemic lupus erythematosus. Arthritis Rheum 1997;40 10.1002/art.1780400928 9324032

[12] Petri M, Orbai A-M, Alarcón GS, et al Derivation and validation of the systemic lupus international collaborating clinics classification criteria for systemic lupus erythematosus. Arthritis Rheum 2012;64:2677–86. 10.1002/art.34473 22553077 PMC3409311

[13] Gladman DD, Ibanez D, Urowitz MB Systemic lupus erythematosus disease activity index 2000. J Rheumatol 2002;29:288–91. 11838846

[14] Isenberg DA, Rahman A, Allen E, et al BILAG 2004. Development and initial validation of an updated version of the British Isles lupus Assessment Group's disease activity index for patients with systemic lupus erythematosus. Rheumatology 2005;44:902–6. 10.1093/rheumatology/keh624 15814577

[15] Gladman DD, Urowitz MB, Slonim D, et al Evaluation of predictive factors for neurocognitive dysfunction in patients with inactive systemic lupus erythematosus. J Rheumatol 2000;27:2367–71. 11036831

[16] Zigmond AS, Snaith RP The hospital anxiety and depression scale. Acta Psychiatr Scand 1983;67:361–70. 10.1111/j.1600-0447.1983.tb09716.x 6880820

[17] Beck AT, Steer RA, Brown GK Manual for the Beck depression Inventory-II. San Antonio, Texas: Psychological Corporation, 1996.

[18] Williams JBW, Kobak KA Development and reliability of a structured interview guide for the Montgomery Asberg depression rating scale (sigma). Br J Psychiatry 2008;192:52–8. 10.1192/bjp.bp.106.032532 18174510

[19] Penner IK, Raselli C, Stöcklin M, et al The fatigue scale for motor and cognitive functions (FSMC): validation of a new instrument to assess multiple sclerosis-related fatigue. Mult Scler 2009;15:1509–17. 10.1177/1352458509348519 19995840

[20] CANTAB CCArr: CANTAB(R) [Cognitive assessment software], 2016 Available: www.cantab.com

[21] Friston K Ten ironic rules for non-statistical reviewers. NeuroImage 2012;61:1300–10. 10.1016/j.neuroimage.2012.04.018 22521475

[22] Yee C-S, Cresswell L, Farewell V, et al Numerical scoring for the classic BILAG index. Rheumatology 2010;48:1548–52. 10.1093/rheumatology/kep183 PMC2777486 19779027

[23] Benedek M, Jauk E, Beaty RE, et al Brain mechanisms associated with internally directed attention and self-generated thought. Scientific Reports 2016;6 10.1038/srep22959 PMC4785374 26960259

[24] Rombouts SA, Scheltens P, Machielson WC, et al Parametric fMRI analysis of visual encoding in the human medial temporal lobe. Hippocampus 1999;9:637–43. 10.1002/(SICI)1098-1063(1999)9:6<637::AID-HIPO4>3.0.CO;2-V 10641756

[25] Mattfeld AT, Whitfield-Gabrieli S, Biederman J, et al Dissociation of working memory impairments and attention-deficit/hyperactivity disorder in the brain. Neuroimage Clin 2016;10:274–82. 10.1016/j.nicl.2015.12.003 26900567 PMC4723732

[26] Wiseman SJ, Bastin ME, Hamilton IF, et al Fatigue and cognitive function in systemic lupus erythematosus: associations with white matter microstructural damage. a diffusion tensor MRI study and meta-analysis. Lupus 2016. 10.1177/0961203316668417 PMC5374047 27687026

[27] Charidimou A, Meegahage R, Fox Z, et al Enlarged perivascular spaces as a marker of underlying arteriopathy in intracerebral haemorrhage: a multicentre MRI cohort study. Journal of Neurology, Neurosurgery &amp. Psychiatry 2013;84:624–9. 10.1136/jnnp-2012-304434 PMC3905629 23412074

[28] Andrews-Hanna JR, Smallwood J, Spreng RN The default network and self-generated thought: component processes, dynamic control, and clinical relevance. Annals of the New York Academy of Sciences 2014;1316:29–52. 10.1111/nyas.12360 24502540 PMC4039623

[29] Roiser JP, Sahakian BJ Hot and cold cognition in depression. CNS Spectrums 2013;18:139–49. 10.1017/S1092852913000072 23481353

[30] Arnone D, McKie S, Elliott R, et al Increased amygdala responses to sad but not fearful faces in major depression: relation to mood state and pharmacological treatment. Am J Psychiatry 2012;169:841–50. 10.1176/appi.ajp.2012.11121774 22854930

[31] Gollan JK, McCloskey M, Hoxha D, et al How do depressed and healthy adults interpret nuanced facial expressions? J Abnorm Psychol 2010;119:804–10. 10.1037/a0020234 20939654 PMC3805828

[32] Leppänen JM Emotional information processing in mood disorders: a review of behavioral and neuroimaging findings. Curr Opin Psychiatry 2006;19:34–9. 10.1097/01.yco.0000191500.46411.00 16612176

[33] White DA, Myerson J, Hale S How cognitive is psychomotor slowing in depression? Evidence from a meta-analysis. Aging, Neuropsychology, and Cognition 1997;4:166–74. 10.1080/13825589708256645

[34] Levy R, Friedman HR, Davachi L, et al Differential activation of the caudate nucleus in primates performing spatial and nonspatial working memory tasks. J Neurosci 1997;17:3870–82. 10.1523/JNEUROSCI.17-10-03870.1997 9133405 PMC6573717

[35] Mak A, Ren T, Fu EH-yun, et al A prospective functional MRI study for executive function in patients with systemic lupus erythematosus without neuropsychiatric symptoms. Semin Arthritis Rheum 2012;41:849–58. 10.1016/j.semarthrit.2011.11.010 22221909

[36] Kozora E, Thompson LL, West SG, et al Analysis of cognitive and psychological deficits in systemic lupus erythematosus patients without overt central nervous system disease. Arthritis & Rheumatism 1996;39:2035–45. 10.1002/art.1780391213 8961909

[37] Sweet JJ, Doninger NA, Zee PC, et al Factors influencing cognitive function, sleep, and quality of life in individuals with systemic lupus erythematosus: a review of the literature. Clin Neuropsychol 2004;18:132–47. 10.1080/13854040490507244 15595365

[38] Zunszain PA, Hepgul N, Pariante CM Inflammation and depression. Curr Top Behav Neurosci 2013;14:135–51. 10.1007/7854_2012_211 22553073

[39] Mackay M, Bussa M, et al Differences in regional brain activation patterns assessed by functional magnetic resonance imaging in patients with systemic lupus erythematosus stratified by disease duration. Mol Med 2011;17:1–56. 10.2119/molmed.2011.00185 PMC3321819 21953419

[40] Lenehan ME, Summers MJ, Saunders NL, et al Does the Cambridge automated neuropsychological test battery (Cantab) distinguish between cognitive domains in healthy older adults? Assessment 2016;23:163–72. 10.1177/1073191115581474 25882162

[41] Kirchner WK Age differences in short-term retention of rapidly changing information. J Exp Psychol 1958;55:352–8. 10.1037/h0043688 13539317

[42] Ekman P, Friesen WV Measuring facial movement. J Nonverbal Behav 1976;1:56–75. 10.1007/BF01115465

[43] Patankar TF, Mitra D, Varma A, et al Dilatation of the Virchow-Robin space is a sensitive indicator of cerebral microvascular disease: study in elderly patients with dementia. AJNR Am J Neuroradiol 2005;26:1512–20. 15956523 PMC8149063

