# Supplementary material for: Altered cognitive function in systemic lupus erythematosus and associations with inflammation and functional and structural brain changes
Source: Ann Rheum Dis. 2019 Apr 12;78(7):934–40. doi: 10.1136/annrheumdis-2018-214677 (PMC6585286; doi:10.1136/annrheumdis-2018-214677)
Supplement: Supplementary data [file annrheumdis-2018-214677supp001.docx]

# SUPPLEMENTARY DATA

**Serological analysis**

IL-6, BLyS, VEGF and VCAM-1 were all measured using DuoSet ELISA development kits from R&D Systems (Abingdon, UK). hs-CRP was measured by an in-house sandwich ELISA method using anti-human CRP antibodies, calibrators and controls from Abcam (Cambridge, UK) by the specialist assay unit at MFT (P.P.).

**CANTAB® scoring**

Each task has multiple outcome measures but the measures used in this study were as follows. For PAL; total errors (adjusted), VRM; free recall-total correct, ERT; average percentage correct-total and overall mean response latency-total (ms), RVP; total hits, OTS; Mean choices to correct, and SWM; between errors[20].

**MRI methods**

The n-back and FERT images were acquired using a whole-brain dual echo T2*-weighted sequence with TR=2.3s, TE1/TE2=12ms/35ms, in-plane-resolution=3mm x 3mm and 28 slices of 3.8mm thickness. Total scan time for n-back was 6mins 53s (180 volumes) and for FERT was 7mins 21s (192 volumes).

T2-weighted 3D FLAIR were taken with a TR of 4800ms, TE=256ms, TI=1650ms and 180 isotropic slices of 0.83mm over 7mins 26s. The MP-RAGE sequence produced T1-weighted images with a TR of 8.4ms, TE=3.8ms and 180 isotropic slices of 0.83ms over 5mins 43s.

**n-back**

The functional n-back task was developed from a well-established task by Kirchner[41], the n-back examines attention and working memory. Participants watched a series of individual letters flash on a screen and were required to press a button in response to certain stimuli. The task involved three conditions, referred to as, 0-back, 1-back and 2-back. 0-back was the easiest and 2-back the most challenging. For each condition 13 different letters were presented one at a time. In the 0-back condition participants had to press the button if they saw an “X”. For the 1-back condition participants had to press the button when the same letter appeared consecutively. Finally, the 2-back condition required participants to press when the letter presented was the same as the one before last, for example a V, followed by a T, followed by a V. The 0-back condition examines attention and the 1 and 2-back conditions working memory. There were 3 blocks and each block consisted of the 1-back and 2-back conditions presented once each interspersed with 2 presentations of the 0-back condition. After each block there was a 29.5s rest period (online supplementary figure S1).

**FERT**

The functional FERT task consisted of a series of faces originally developed by Ekman and Friesen presented to the participants to assess emotional processing[42]. Participants were asked to indicate, by using a button box, if the face they saw was male or female. They were not told that the task was examining emotional processing. The participants were shown faces displaying three different emotions at 100% intensity – happiness, sadness, and fear – as well as a neutral face. Six different images (three male and three female in a pseudo-random order) of each emotion were shown followed by six different neutral faces. After each emotion was shown once (one block) the participant was given a 21s break where just a fixation cross remained on the screen. There were three blocks in total (online supplementary figure S2).

**fMRI analyses**

fMRI data were analysed in SPM12. The functional image data underwent realignment, coregistration, segmentation, normalisation and smoothing as part of the preprocessing stage. During realignment the rigid body transformation method was used to correct for minor head motion. Co-registration was used to align the structural image within the mean functional image space. The structural image was segmented into six components and then normalised into standard space using the standard “European brains” template. The warping applied to the structural image was then applied to the realigned functional images. Finally, smoothing was conducted by applying a Gaussian kernel with a full width half maximum of 8 8 8. The participant data was then checked for motion artefacts using the “ART” toolbox and motion and global mean intensity outliers were reported. Participants with >20% of images showing motion artefacts were excluded.

Individual participant data were then modelled using a general linear model approach. This produced contrast images (the differences in BOLD response during the different conditions of the fMRI tasks).

Group region of interest (ROI) analyses were undertaken using these contrast images. ROIs were obtained using clusters identified for the positive and negative main effects of the tasks at *p*=0.001. Mean BOLD response values were extracted per participant from clusters with an extent threshold of Family Wise Error-corrected *p(FWEc)*<0.05. These values were then analysed in SPSS 22 for differences between the participant groups using independent t-tests.

The FLAIR and T1 images were examined by a neuroradiologist (A.J.) and scores for periventricular hyperintensities (PVH), deep white matter hyperintensities (DWMH) and abnormalities in the brainstem and basal ganglia were reported. The scores were calculated based on a modified version of the Schelten scale. Reports of the perivascular spaces (Virchow-Robin spaces) in the basal ganglia (BG-VRS) and centrum semiovale (CSO-VRS) were also provided, details of these scoring systems are stated elsewhere[43]. Chi-square analysis of the BG-VRS and CSO-VRS scores were undertaken.

## Supplementary figures

**Figure S1. n-back task description:** There were three different conditions (0-back, 1-back and 2-back). Participants were presented with the instructions for one condition at a time followed by 13 letters. The order of the conditions for the first block was 0-, 1-, 0- and 2-back, followed by a rest, the second block 0-, 2-, 0-, and 1-back, followed by a rest and then the final block 0-, 1-, 0-, and 2-back.

n-back: Get ready

c...

+

Thank you

**→**

**→**

**→**

Blank screen: 0.5s Letter stimuli: 1.5s Rest: 29.5s End: 10s

Instructions: 3.5s

Press when see X

0-back

Press when letter same as last

1-back

Press when letter same as one before last

2-back

**x3 →**

**x13 →**

**x4 →**

Block

**Figure S2. FERT description:** Participants were presented with 6 different faces displaying the same emotion. There were 4 emotions: Neutral (N), happiness (H), sadness (S) and fear (F). In block 1 participants saw 6 faces of N, H, N, S, N, F followed by a rest. Block 2 showed 6 faces of N, S, N, F, N, H followed by a rest. Finally block 3 showed 6 faces of N, F, N, H, N, S and then the end of the task.

1 male, 2 female


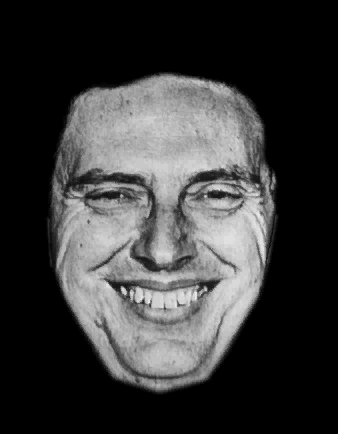


+

Thank you

**→**

**→**

Blank screen: 0.5s Emotion: 3s Rest: 21s End: 10s

**x6 →**

**x6 →**

**X3 →**

Block

## Supplementary tables

Table S1 Correlations within the SLE group for the depression, fatigue and IQ variables

| **Assessment** | **FSMC-Motor** | **FSMC-Cognition** | **Years in education** |
| --- | --- | --- | --- |
| MADRS | *r*=0.57, *p*=0.001 | *r*=0.48, *p*=0.007 | *r*=-0.44, *p*=0.015 |
| HADS-D | *r_s_*=0.67, *p*<0.001 | *r_s_*=0.58, *p*=0.001 | *r_s_*=-0.46, *p*=0.005 |
| BDI-II | *r*=0.63, *p*<0.001 | *r*=0.63, *p*<0.001 | *r*=-0.48, *p*=0.004 |
| WTAR (IQ) | *n/a* | *n/a* | *r*=0.39, *p*=0.027 |
| *MADRS: Montgomery Asberg Depression Rating Scale; HADS-D: Hospital Anxiety and Depression Scale – Depression score; BDI-II: Beck’s Depression Inventory-II; WTAR: Weschler Test of Adult Reading; FSMC: Fatigue Scale for Motor and Cognitive functions* | | | |

Table S2 Median brain abnormality scores for the SLE and HC groups

| **Result** | **SLE** | **HC** |
| --- | --- | --- |
|  | **Median (Lower quartile, upper quartile)** | |
| Periventricular hyperintensities (PVH) total | 0 (0, 0) | 0 (0, 0) |
| Deep white matter hyperintensities (DWMH) total | 0 (0, 3) | 0 (0, 3) |
| Brainstem total | 0 (0, 0) | 0 (0, 0) |
| Basal ganglia total | 0 (0, 0) | 0 (0, 0) |

Table S3 Percentage of participants per group that scored in each of the CSO-VRS categories

| **Group** | **Centrum semiovale Virchow Robin Space (CSO-VRS)** | | | |
| --- | --- | --- | --- | --- |
|  | **0** | **1** | **2** | **3** |
| **SLE** | 57 | 26 | 13 | 4 |
| **HC** | 100 | 0 | 0 | 0 |
| *0 = none*  *1= less than five per side*  *2 = More than five on one or both sides*  *3 = More than 20 per side* | | | | |

Table S4 Percentage of participants per group that scored in each of the BG-VRS categories

| **Group** | **Basal Ganglia Virchow Robin Space (BG-VRS)** | | | | | |
| --- | --- | --- | --- | --- | --- | --- |
|  | **0** | **1** | **2** | **3** | **4** | **5** |
| **SLE** | 26 | 4 | 17 | 17 | 30 | 0 |
| **HC** | 40 | 7 | 23 | 27 | 3 | 0 |
| *0 = VRS present only in the substantia innominata and fewer than five VRSs on either side*  *1= VRS only in the substantia innominata or more than five dilated VRS on either side*  *2 = fewer than five in the lentiform nucleus on either side*  *3 = five to 10 VRSs in the lentiform or fewer than five in the caudate nucleus on either side*  *4 = more than 10 in the lentiform nucleus and fewer than five in the caudate nucleus on either side*  *5 = more than 10 in lentiform nucleus and more than five in the caudate nucleus on either side* | | | | | | |

Table S5 Behavioural results from the fMRI n-back task, SLE vs HC

| **Variable** | | **SLE (n=23)**  **Mean (SD)**  **Median (LQ, UQ)** | **HC (n=29)**  **Mean (SD)**  **Median (LQ, UQ)** | ***p*-value** |
| --- | --- | --- | --- | --- |
| Correct responses | 0-back  (total =18) | 18 (17, 18) | 18 (18, 18) | ***p*=0.008** |
|  | 1-back  (total = 9) | 9 (9, 9) | 9 (9, 9) | *p*=0.260 |
|  | 2-back  (total =9) | 8 (6, 8) | 8 (7, 8.25) | *p*=0.223 |
| Response times  (correct answers only) (s) | 0-back | 0.55 (0.48, 0.62) | 0.51 (0.49, 0.55) | *p*=0.149 |
|  | 1-back | 0.62 (0.53, 0.74) | 0.56 (0.50, 0.59) | ***p*=0.019** |
|  | 2-back | 0.75 (0.62, 0.83) | 0.60 (0.56, 0.72) | ***p*=0.025** |
| Overall incorrect responses | | 1 (0, 1) | 1 (0, 1) | *p*=0.733 |

Table S6 Behavioural results from the FERT task (correct responses refer to participants correctly identifying the gender of the face represented not the emotion) – SLE vs HC

| **Variable** | **Emotion expressed** | **SLE (n=23)**  **Mean (SD)**  **Median (LQ, UQ)** | **HC (n=29)**  **Mean (SD)**  **Median (LQ, UQ)** | ***p*-value** |
| --- | --- | --- | --- | --- |
| Total number of genders correctly identified | All emotions  (max. score=108) | 106 (101.75, 107) | 106 (103.50, 108) | *p*=0.434 |
|  | Neutral  (max. score=54) | 52.50 (49.75, 53) | 53 (52, 54) | *p*=0.212 |
|  | Happiness  (max. score=18) | 18 (17.75, 18) | 18 (17.50, 18) | *p*=0.858 |
|  | Sadness  (max. score=18) | 18 (17, 18) | 18 (17, 18) | *p*=0.677 |
|  | Fear  (max. score=18) | 18 (17, 18) | 18 (18, 18) | *p*=0.140 |
| Average response times (s) | All emotions | 0.97 (0.19) | 0.89 (0.16) | *p*=0.125 |
|  | Neutral | 0.95 (0.17) | 0.89 (0.17) | *p*=0.177 |
|  | Happiness | 0.97 (0.81, 1.04) | 0.84 (076, 1.03) | *p*=0.084 |
|  | Sadness | 0.98 (0.20) | 0.89 (0.16) | *p*=0.068 |
|  | Fear | 0.93 (0.83, 1.19) | 0.83 (0.75, 1.05) | *p*=0.102 |
| Average response time for only correctly identified genders (s) | All emotions | 0.95 (0.18) | 0.88 (0.15) | *p*=0.123 |
|  | Neutral | 0.93 (0.16) | 0.87 (0.15) | *p*=0.168 |
|  | Happiness | 0.94 (0.81, 1.05) | 0.84 (0.76, 1.03) | *p*=0.077 |
|  | Sadness | 0.91 (0.85, 1.08) | 0.83 (0.76, 1.00) | ***p*=0.035** |
|  | Fear | 0.94 (0.82, 1.08) | 0.83 (075, 1.05) | *p*=0.154 |
